# Diagnosing underdetermination in stable isotope mixing models

**DOI:** 10.1371/journal.pone.0257818

**Published:** 2021-10-01

**Authors:** Yutaka Osada, Jun Matsubayashi, Ichiro Tayasu

**Affiliations:** 1 Fisheries Resources Institute, Japan Fisheries Research and Education Agency, Yokohama, Kanagawa, Japan; 2 Research Institute for Humanity and Nature, Kyoto, Japan; University of Hyogo, JAPAN

## Abstract

Stable isotope mixing models (SIMMs) provide a powerful methodology for quantifying relative contributions of several sources to a mixture. They are widely used in the fields of ecology, geology, and archaeology. Although SIMMs have been rapidly evolved in the Bayesian framework, the underdetermination of mixing space remains problematic, i.e., the estimated relative contributions are incompletely identifiable. Here we propose a statistical method to quantitatively diagnose underdetermination in Bayesian SIMMs, and demonstrate the applications of our method (named *β*-dependent SIMM) using two motivated examples. Using a simulation example, we showed that the proposed method can rigorously quantify the expected underdetermination (i.e., intervals of *β*-dependent posterior) of relative contributions. Moreover, the application to the published field data highlighted two problematic aspects of the underdetermination: 1) ordinary SIMMs was difficult to quantify underdetermination of each source, and 2) the marginal posterior median was not necessarily consistent with the joint posterior peak in the case of underdetermination. Our study theoretically and numerically confirmed that *β*-dependent SIMMs provide a useful diagnostic tool for the underdetermined mixing problem. In addition to ordinary SIMMs, we recommend reporting the results of *β*-dependent SIMMs to obtain a biologically feasible and sound interpretation from stable isotope data.

## Introduction

In animal ecology, the development of a methodology for quantifying trophic interactions between consumers and their dietary sources has a long history [1, 2]. Stable isotope mixing models (SIMMs) are popular statistical tools not only for ecologists to estimate the relative contribution of each dietary source to consumers based on isotopic signatures [3, 4], but also used in the other fields such as climatology, oceanography, sedimentology and archaeology [5–7]. Using a Bayesian framework, the applicability of SIMMs to complex isotopic mixing spaces of realistic systems has rapidly improved [8]. The improvements include the incorporation of measurement errors [9], isotopic correlations [10], element concentrations [11, 12], dietary routings [13], additional residual errors of unknown sources [12], and hierarchical structures of consumer populations [14] and food-webs [15]. More recently, most of them are applicable as an open-source program [16].

However, the considerable advances in SIMMs have made it tempting to overlook a fundamental issue in mixing models, the underdetermined mixing problem [7, 16–19] (see S1 Appendix in S1 File). As the terminology suggests, this is a statistical problem related to the underdetermination of estimates, originating from the analysis of many dietary sources against few elements and/or problematic isotopic geometries (e.g., three or more sources are arranged on the same line) in mixing spaces [17]. Let consider a simple example of underdetermined mixing problem for Bayesian models. In this example, we are interested in a consumer species, which has two candidate dietary sources with their relative contributions, *θ* and 1−*θ* (*θ* can be either 1.0, 0.5 or 0.0 for simplicity). For sound mixing spaces, the unique relative contributions of these sources have the maximal posterior probability, e.g., *P*(*θ* = 0.5)>*P*(*θ* = 1.0)≥*P*(*θ* = 0.0). Statistically, such relative contributions are referred to as being identifiable (or estimable). On the other hands, the underdetermined mixing problem has two or more relative contributions with the maximal posterior probability, e.g., *P*(*θ* = 0.5) = *P*(*θ* = 1.0)≥*P*(*θ* = 0.0). Importantly, this problem may result in the inappropriate interpretation and wrong secondary use of SIMM results; in the case of underdetermination, SIMM results cannot be summarized only by a representative value (mean, median or mode) of the marginal posterior distribution.

**Fig 1:**
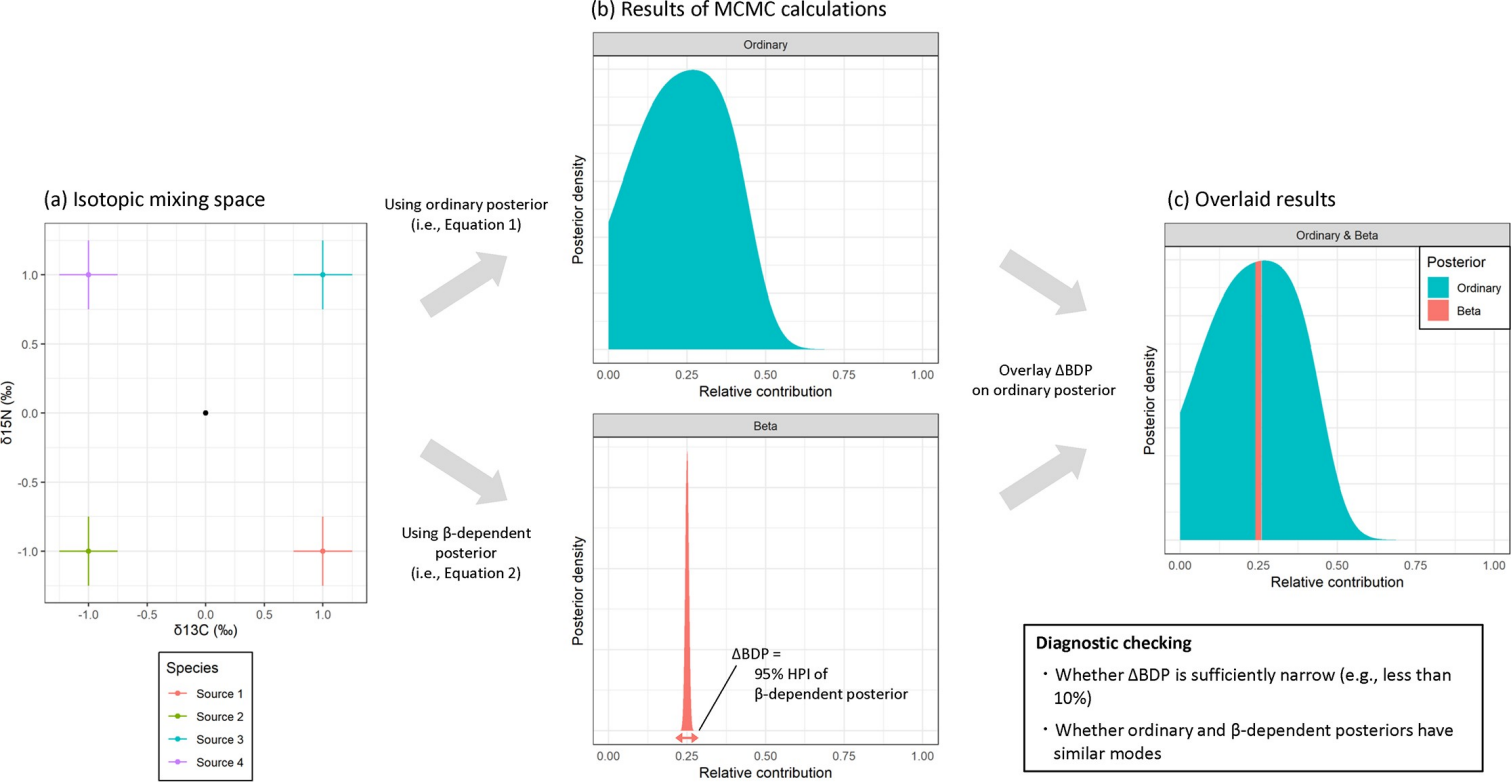
Proposed workflow for diagnostic checking of the underdetermined problem. First, we calculate the posteriors of both ordinary and *β*-dependent SIMM (panel b) models from isotopic mixing space (panel a). Second, ΔBDP (i.e., 95% highest posterior interval of *β*-dependent posterior) is overlaid on the ordinary posterior (panel c), which make us easy diagnostic checking.

**Fig 2:**
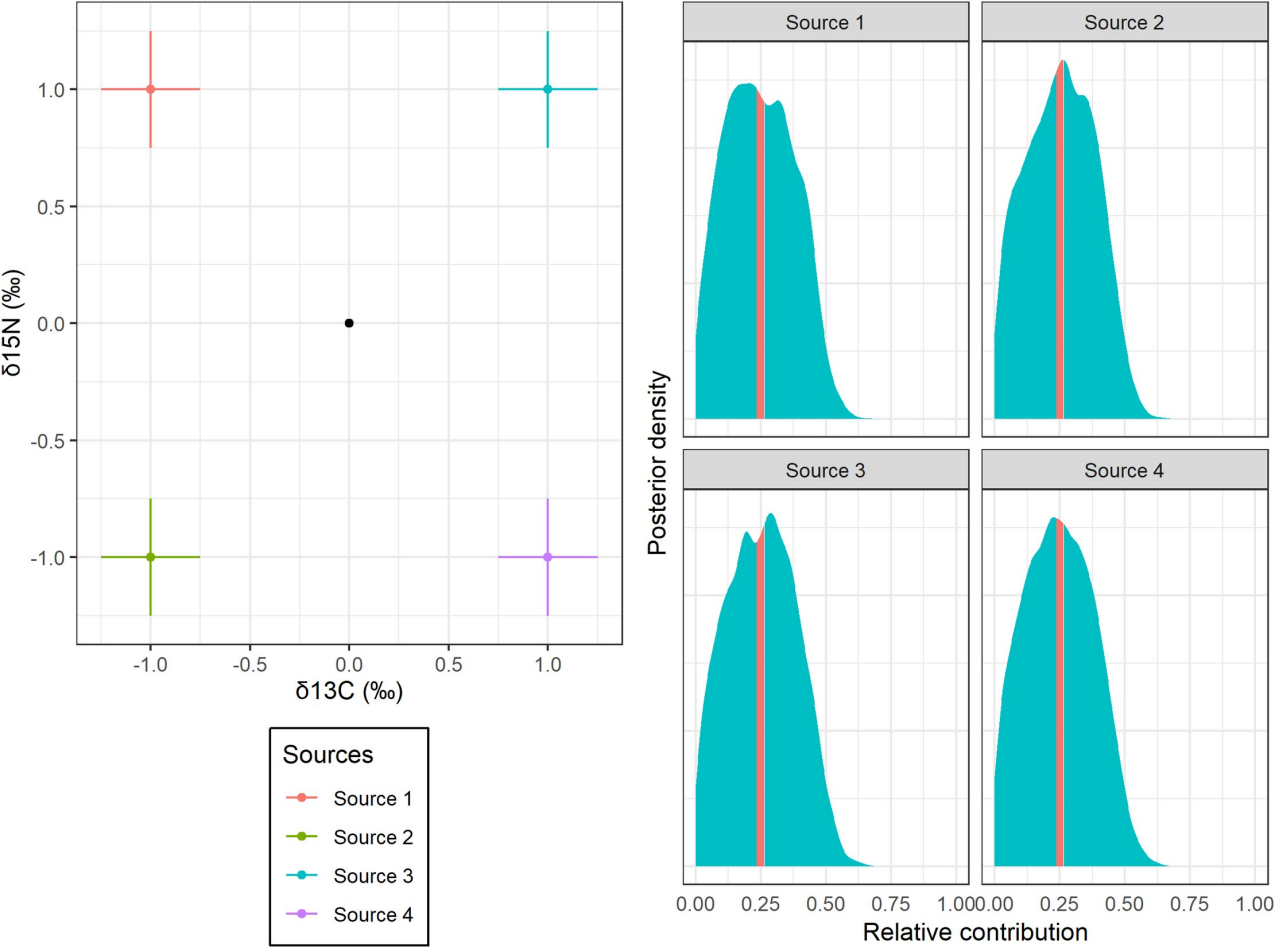
Isotopic mixing space and SIAR results of a toy simulation. The mixing space (left panel) represents the isotopic value of an individual consumer (a black circle), and the means and standard deviations of isotopic values of four sources (colored circles and bars). The posterior distribution of each relative contribution (right panel) was calculated from ordinary (blue area) and *β*-dependent SIAR model (red area). The theoretical relative contribution is represented in Fig 1C.

**Fig 3:**
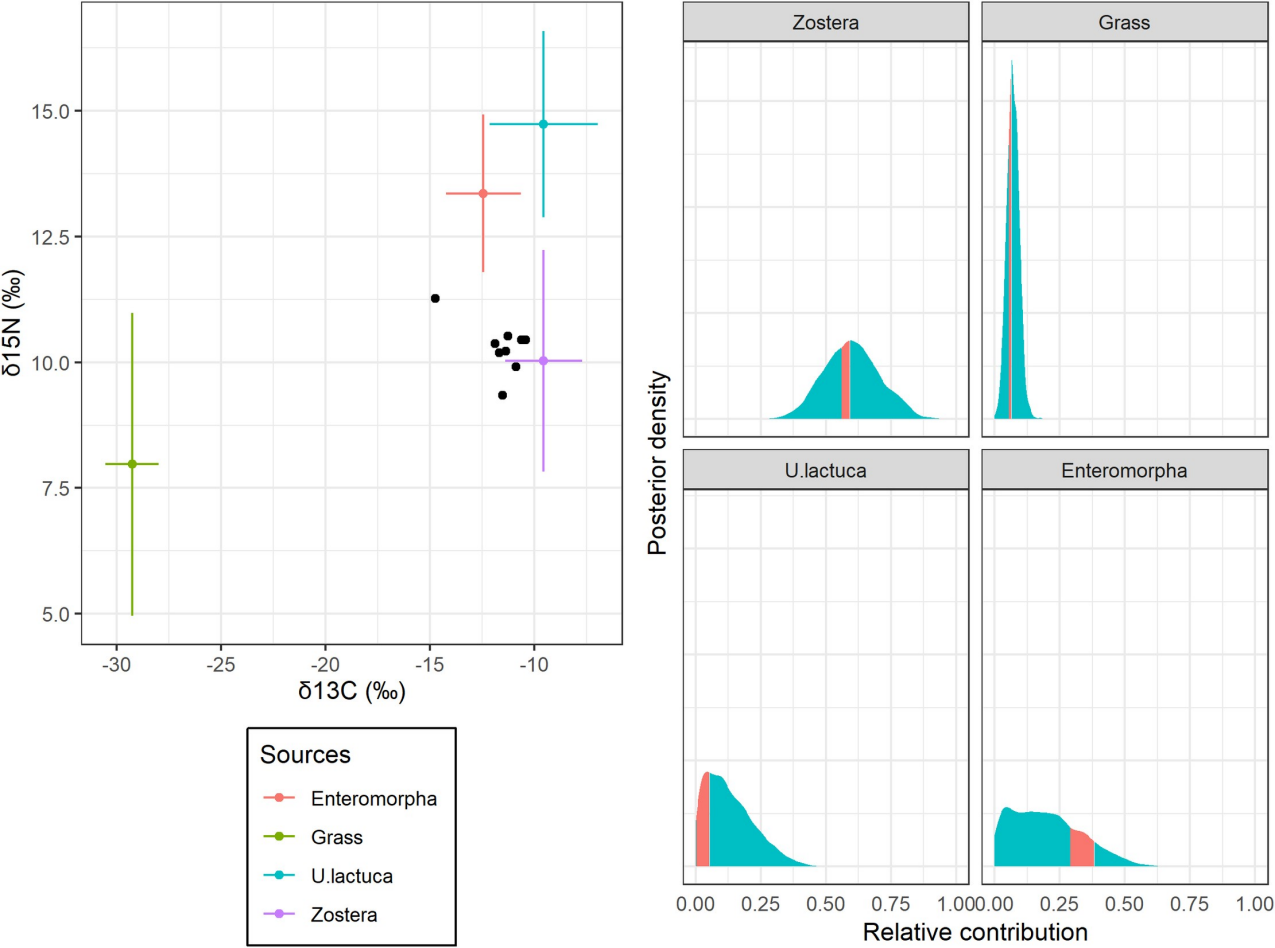
Isotopic mixing space and SIAR results of published Brent geese data. The mixing space (left panel) represents the fractionally corrected isotopic values of individual consumers (black circles), and the means and standard deviations of isotopic values of four sources (colored circles and bars). The posterior distribution of each relative contribution was calculated from ordinary (blue area) and *β*-dependent SIAR model (red area).

The underdetermination is a general statistical problem but particularly notorious for SIMMs, in which increasing the number of elements, rather than the number of isotope samples, is needed to improve the identifiability of relative contributions. Previous studies proposed several methods to diagnose this problem (e.g., graphical checking [17], posterior correlation [18], posterior multi-modality [18] and normalized source polygon area [19]). However, these methods are unsuitable for diagnosing complex mixing spaces because their diagnostic signals are sensitive to various sources of data uncertainty. Here we present an alternative method to quantitatively diagnose the underdetermination for existing Bayesian SIMMs (Fig 1). In this paper, we explained how our method obtains the intervals of joint posterior peaks as an accurate diagnostic of underdetermination, and demonstrated its applications to two motivated examples; a simple toy simulation [17] (Fig 2) and a published field dataset for Brent geese (*Branta bernicla*) [12, 20] (Fig 3). The proposed method uses a simple, general statistical framework (i.e., *β*-dependent posterior probability), and thus achieves accurate diagnosability and broad applicability to all Bayesian SIMMs.

## Materials and methods

### Diagnosing underdetermination in SIMMs

Since SIMM is one of Bayesian statistical models, its posterior probability, *p*(***θ***|***φ***, ***X***), is derived from a prior, *π*(***θ***), and a data probability, *p*(***X***|***θ***, ***φ***), as follows:

p(θ|φ,X)=p(X|θ,φ)π(θ)/∫p(X|θ,φ)π(θ)dθ, (1)

where ***θ*** is a vector of estimated quantities (e.g., relative contributions), ***φ*** is a vector of user-given quantities, and ***X*** is a vector of isotopic data. As an example of Eq (1), we described the model of SIAR [12] in S2 Appendix in S1 File. To diagnose underdetermination, we introduced a *β*-dependent posterior probability:

$$p_{\beta }(\theta |\varphi ,X)={p(X|\theta ,\varphi )}^{\beta }\;{\pi (\theta )}^{\beta }/\int {p(X|\theta ,\varphi )}^{\beta }\;{\pi (\theta )}^{\beta }\;d\theta .$$ (2)

*β*-dependent posterior probability with *β* = 1 reduces to the ordinary posterior probability. For large *β* (→ ∞), the posterior probability of estimates other than the join posterior peak become zeros (see S3 Appendix in S1 File for this proof). Thus, *β*-dependent posterior distributions with arbitrarily large *β* correspond to the distributions of the joint posterior peaks. An intrinsic issue of the underdetermined problem is low identifinability among the relative contributions of two or more sources, and thereby provides wide intervals of *β*-dependent posterior distributions (BDP). Importantly, the model complexity of SIMMs does not influence diagnosability of our method. In this paper, we used the ΔBDP (the BDP width of 95% highest density probability interval) as a quantitative diagnostic for underdetermination. The ΔBDP will be near zero when the underdetermined problem does not exist in the focal mixing space. Note that *β* has little influence on ΔBDP if the *β* is sufficiently large (e.g., > 500).

Our method is non-Bayesian despite adoption of the full Bayesian setup for standard Markov chain Monte Carlo calculations. Because it works just as the device to numerically obtain the joint posterior peaks (i.e., BDP), simultaneous calculations of the ordinary posterior are needed to interpret the whole results of Bayesian SIMMs (Fig 1). Note that our method is closely related to previous established methods of obtaining maximum likelihood estimates (MLE) [21, 22] (also see S3 Appendix in S1 File). The difference between our method and their MLE methods is whether priors are exponentiated or not.

### Motivated examples

We provide two motivated examples. The first example is a two-isotope simulation in which the consumer signature is at the centroid of four source signatures (Fig 2; see S4 Appendix in S1 File for simulation details). This simulation was used to diagnose the underdetermination by previous diagnostic methods [17, 18]. It also helps to confirm the theoretical validity of our method: the BDP of each relative contribution theoretically becomes 23.9–26.0% in our setting. The second motivated example is a set of published field data collected to investigate the relative contributions of four different dietary sources (two seagrasses, green algae, and terrestrial grasses) to Brent geese by using carbon and nitrogen stable isotope ratios [12, 20] (Fig 3).

In these examples, we estimated relative contributions of sources to the mixture using ordinary SIAR and *β*-dependent SIAR (i.e., SIAR with *β*-dependent posterior probability). In the latter, we set *β* = 1,000 because sufficiently large *β* qualitatively unchanged the results (see S3 Appendix in S1 File). As described above, we used ΔBDP to diagnose underdetermination for each source relative contribution. We also conducted correlation analyses between the estimated relative contribution of a source and the others (S5 Appendix in S1 File). The mean determination coefficient (${\bar{r}}^{2}$) is considered as another indicator of underdetermination [18], which will approach one if underdetermination exists. The posterior multi-modality was not used as an indicator of underdetermination because our examples exhibit no multi-modality. To obtain the posterior distributions of both SIARs numerically, the Gibbs sampling and importance sampling was implemented using R 4.1.1 [23] (our implementation is available as an R package ‘siarbeta’ at https://github.com/yutakaos/archives/tree/master/simm/siarbeta). We assigned uninformative or vague priors for all parameters according to previous studies [12, 16].

### Different error structure parameterizations

The recent publications [16, 24] compared the model performance between different error structure parameterizations. To investigate the influence of error structure parameterizations on underdetermination, we conducted an additional analysis for published geese data using the Stock’s parameterization model (i.e., Eq 4 in [24]). The details of model formulations and results are described in S6 Appendix in S1 File.

## Results

In the toy simulation, the estimated relative contributions of sources to the mixture were moderately correlated (${\bar{r}}^{2}$ = 0.676, 0.674, 0.675 and 0.676; Table 1, S1 Fig in S1 File). On the other hand, the *β*-dependent SIAR exhibited relatively narrow BDP for all sources (23.5–26.4% for all sources; Table 1, Fig 2). These intervals were much the same as the expected theoretical intervals (i.e., 23.9–26.0%).

**Table 1:** Estimated relative contributions (%) of dietary sources for two motivated examples.

	medians	95% CI	${\bar{r}}^{2}$	BDP	ΔBDP
**Toy simulation**					
Source A	24.1	[1.8, 48.9]	0.676	[23.5, 26.4]	2.9
Source B	25.5	[2.0, 49.1]	0.674	[23.5, 26.4]	2.9
Source C	25.4	[2.1, 49.9]	0.675	[23.5, 26.4]	2.9
Source D	24.6	[2.0, 49.4]	0.676	[23.5, 26.4]	2.9
**Field data**					
*Zostera*	59.7	[40.0, 81.0]	0.186	[55.9, 59.2]	3.3
Terrestrial grasses	6.9	[2.5, 11.7]	0.122	[5.3, 6.3]	1.0
*Ulva lactuca*	11.3	[0.6, 34.7]	0.133	[0.0, 5.4]	5.4
*Enteromorpha*	18.5	[0.9, 48.5]	0.330	[29.1, 38.3]	9.2

The medians, 95% credible intervals (CI) and mean determination coefficient (${\bar{r}}^{2}$) are calculated from ordinary SIAR. The BDPs and their widths (ΔBDP) are calculated from *β*-dependent SIAR. Note that the expected BDPs of our toy simulation are [23.9, 26.0]. In all analyses, we set *β* = 1,000 for *β*-dependent SIAR.

Using the published geese data, the estimated relative contributions of dietary items were weakly correlated (${\bar{r}}^{2}$ = 0.186, 0.122, 0.133 and 0.330; Table 1, S2 Fig in S1 File). However, ΔBDP varies substantially among different sources. Terrestrial grasses had a narrow BDP (5.3–6.3%), while *Enteromorpha* spp. had a relatively wide BDP (29.1–38.3%). The BDP of *Zostera* and *Ulva lactuca* were intermediate (55.9–59.2% and 0.0–5.4%, respectively). Interestingly, all the marginal posterior medians fell outside the BDP (Table 1, Fig 3). We also found that changing error structure parameterization from SIAR to Stock’s model leads to wider ΔBDP but improves the consistency between ordinary and beta-dependent marginal posteriors (S1 Table, S3 Fig in S1 File).

## Discussion

In our simulation, our method provides the expected theoretical intervals for relative contributions of each source to the mixture. This confirms that *β*-dependent posterior distributions can quantitatively diagnose the underdetermined mixing problem. Because *β*-dependent posterior probability can be applied even for complex mixing systems, our method balances accurate diagnosability and broad applicability. This is the biggest advantage over previous methods such as graphical checking, posterior correlation, posterior multi-modality and normalized source polygon area [17–19].

Using the published geese data, we found two problematic aspects of underdetermined mixing problems highlighted by the analysis of *β*-dependent SIMM. First, ΔBDP vary among dietary sources, and the mean determination coefficient from ordinary SIMM cannot quantify the variation (Table 1, Fig 3). This may not be surprising because the BDPs only depend on the geometry of the isotopic mixing space, while the mean determination coefficients depend on both isotopic geometry and other data uncertainties. Second, the marginal posterior medians of ordinary SIMM are necessarily inconsistent with the joint posterior peaks. In this example, all the medians fell outside of the BDPs. This inconsistency was serious for *Enteromorpha* spp. whose relative contribution was most underdetermined (Fig 3). It is explained by failing to approximate marginal posterior distributions to Gaussian distributions [25] due to underdetermination. Furthermore, the slight difference of model structures may unexpectedly influence the underdetermination. We found that the Stock’s error parameterization improved the consistency between ordinary and beta-dependent marginal posteriors at the cost of expanding ΔBDP for published geese dataset. These results emphasize the importance of quantitatively diagnosing underdetermination in SIMMs even in seemingly sound mixing spaces.

The serious underdetermined mixing problem results in high correlation and multi-modality for estimated relative contributions [18]. However, our results showed that these indicators may be a rough diagnostic. For example, *Zostera* had higher mean determination coefficient than those of *Ulva lactuca* (0.186 vs 0.133), but the inverse relationship was observed for ΔBDP (0.8% vs 5.3%). The multi-modality cannot even be detected for our examples. Checking correlation and multi-modality should be recognized as a preliminary tool for diagnosing serious underdetermined problems.

Our method exponentiates not only data probability but also prior probability because our interest is to diagnose underdetermination of posterior probability. Therefore, our method inherits the benefits and limitations of Bayesian methods. Specifically, we can utilize the information from other investigations (e.g., stomach contents analysis) as informative priors, while we can obtain trivial results when data has little information. For Bayesian users, it is important to understand that underdetermined mixing problems are resolved either by improving data probability (i.e., additional isotope elements) or by using informative priors. Future works should aim at determining appropriate priors for underdetermined mixing problems, although it is beyond the scope of this study.

SIMMs are widely used in stable isotope studies to improve the biological interpretation of isotope data. However, misinterpretation may result from underdetermined mixing problems, even in sophisticated field studies. In the case of the Brent geese data, the relative contribution of *Enteromorpha* spp. involved high uncertainty due to underdetermination probably because the isotopic signatures were on the inside of the polygon composed of the other sources. Such problematic isotopic geometries frequently occur in isotopic studies [26–29], requiring a comprehensive discussion of the potential influence of underdetermination on their results. Furthermore, the secondary use of representative estimates for additional analyses is common in isotopic studies [30, 31]. We provide two recommendations for SIMM users. First, the users should report the BDPs in addition to results of ordinary SIMMs, and discuss the influence of underdetermination on their results for sources with moderate ΔBDPs (e.g., ~10%). If interested sources have large ΔBDPs (e.g., 10%~), more isotope elements should be used to remedy underdetermined mixing problems [17]. The allowable underdetermination criterion is context-dependent but for most practical cases, the mixing problem with ΔBDP < 10% may have little influence on the interpretation of SIMM results. Second, the users should not use the marginal posterior medians and modes for secondary use. Instead, we can use the samples from posterior distributions of ordinary or *β*-dependent SIMMs.

Recently, there have been the remarkable developments of biological tracer analysis (e.g., compound-specific stable isotopes of amino acids and fatty acids [32]). These developments should increase the utility and reliability of Bayesian SIMMs. However, we may need to continue struggling the underdetermined problem because our interest will expand to the system with more mixing sources and finer resolution (e.g., species-level to population-level). Hopefully, our method will contribute to sound interpretation and secondary use of isotopic information in many practical settings.

## Supporting information

S1 File (DOCX)

S1 Scripts (ZIP)
