# Supplementary material for: Diagnosing underdetermination in stable isotope mixing models
Source: PLoS One. 2021 Oct 1;16(10):e0257818. doi: 10.1371/journal.pone.0257818 (PMC8486109; doi:10.1371/journal.pone.0257818)
Supplement: S1 File — (DOCX) [file pone.0257818.s001.docx]

**Appendix:**

**Diagnosing underdetermination in stable isotope mixing models**

Y. Osada, J. Matsubayashi and I. Tayasu

**S1. What is the underdetermined mixing problem?**

The basic model structure of all existing SIMMs is a set of simple mass-balance equations (Phillips 2001); in a generic situation where data comprise $N$ consumer individuals with $J$ isotope signatures and $K$ sources, the structure is characterized as

$$X_{ij}=\sum_{k=1}^{K} f_{k}\left( \mu_{jk}+\lambda_{jk} \right)+\varepsilon_{ij} (j=1,\ldots,J) \left( A1 \right)$$

$$\sum_{k=1}^{K} f_{k}=1. \left( A2 \right)$$

Here, $X_{ij}$ is the $j$th isotopic signature of the $i$th consumer individual, $f_{k}$ is the relative contribution of the $k$th dietary source on consumers, $\mu_{jk}$ and $\lambda_{jk}$ are means of the source signature and trophic discrimination factor (TDF) of the $k$th dietary source on the $j$th isotope, and $\varepsilon_{ij}$ is the unexplained residual. Using the above $J+1$ mass-balance equations, SIMMs provide a unique solution for the relative contribution of each source to the mixture only if $K\leq J+1$. Otherwise, SIMMs have an infinite number of solutions. This is not trivial because it means that a representative value (mean, median, or mode) is just one of infinite feasible solutions. The problem is known as the underdetermined mixing problem (Phillips and Gregg 2003; Fry 2013; Semmens et al. 2013; Brett 2014; Stock et al. 2018). In Bayesian SIMMs, there are infinite estimates with the same posterior probability in cases where the underdetermined mixing problem has occurred in mixing spaces.

**S2. An example of Bayesian SIMM: SIAR**

As an example of a Bayesian SIMM, we describe the model structure of SIAR (Stable Isotope Analysis in R; Parnell et al. 2010), one of the most commonly used SIMMs, in the form of Equation (1) in the main text, using $\boldsymbol{\theta}\mathbf{=}\left\{ \boldsymbol{f},\boldsymbol{\sigma} \right\}$ and $\boldsymbol{\varphi}\mathbf{=}\left\{ \boldsymbol{q,\mu,\omega,\lambda,\tau} \right\}$ as follows:

$$p\left( \boldsymbol{X}|\boldsymbol{\theta,\varphi} \right)=p\left( \boldsymbol{X}|\boldsymbol{f},\boldsymbol{q},\boldsymbol{\mu},\boldsymbol{\omega},\boldsymbol{\lambda},\boldsymbol{\tau},\boldsymbol{\sigma} \right)$$

$$=\prod_{i=1}^{N} \prod_{j=1}^{J} N\left( X_{ij} | \frac{\sum_{k=1}^{K} f_{k}q_{jk}\left( \mu_{jk}+\lambda_{jk} \right)}{\sum_{k=1}^{K} f_{k}q_{jk}}, \frac{\sum_{k=1}^{K} {f_{k}}^{2}{q_{jk}}^{2}\left( {\omega_{jk}}^{2}+{\tau_{jk}}^{2} \right)}{\left( \sum_{k=1}^{K} f_{k}q_{jk} \right)^{2}}+{\sigma_{j}}^{2} \right)$$

$$\pi\left( \boldsymbol{\theta} \right)=\pi\left( \boldsymbol{f} \right) \pi\left( \boldsymbol{\sigma} \right)=Dir\left( \boldsymbol{f} | \boldsymbol{a} \right) \left\{ \prod_{j=1}^{J} \mathrm{IG}\left( {\sigma_{j}}^{2} | \alpha_{0}, \alpha_{1} \right) \right\}. \left( A3 \right)$$

In this,

$X_{ij}$ = the $j$th observed isotope signature of the $i$th consumer individual,

$f_{k}$ = relative contribution of the $k$th dietary source,

$\mu_{jk}$ = mean source signature of the $k$th dietary source on the $j$th isotope,

$\lambda_{jk}$ = mean TDF of the $k$th dietary source on the $j$th isotope,

${\omega_{jk}}^{2}$ = source signature variance of the $k$th dietary source on the $j$th isotope,

${\tau_{jk}}^{2}$ = TDF variance of the $k$th dietary source on the $j$th isotope,

$q_{jk}$ = concentration of the $j$th isotope in the $k$th dietary source,

${\sigma_{j}}^{2}$ = additional unexplained variance for the $j$th isotope,

$a_{k}$ = parameters of the Dirichlet prior, and

$\alpha_{0}$ and $\alpha_{1}$ = parameters (shape and scale) of the inverse Gamma prior.

**S3. Behavior of** $\boldsymbol{\beta}$**-dependent posterior distributions for large** $\boldsymbol{\beta}$

This section proves that the posterior probability of estimates other than modes (i.e., the most likely estimates) become zeros when $\beta$-dependent posterior probability has large $\beta\left( \to\infty\right)$. The proof indicates that the joint posterior peaks can be estimated using $\beta$-dependent posterior distribution. In the main text, we defined $\beta$-dependent posterior probability as follows:

$$p_{\beta}\left( \boldsymbol{\theta}|\boldsymbol{\varphi},\boldsymbol{X} \right)={{p\left( \boldsymbol{X}|\boldsymbol{\theta,\varphi} \right)}^{\beta} {\pi\left( \boldsymbol{\theta} \right)}^{\beta}}/{\int{p\left( \boldsymbol{X}|\boldsymbol{\theta,\varphi} \right)}^{\beta} {\pi\left( \boldsymbol{\theta} \right)}^{\beta} d\boldsymbol{\theta}}. \left( A4 \right)$$

Then, Equation (A4) can be deformed below.

$$p_{\beta}\left( \boldsymbol{\theta}|\boldsymbol{\varphi},\boldsymbol{X} \right)$$

$$={\left\{ p\left( \boldsymbol{X}|\boldsymbol{\theta,\varphi} \right) \pi\left( \boldsymbol{\theta} \right) \right\}^{\beta}}/{\int\left\{ p\left( \boldsymbol{X}|\boldsymbol{\theta,\varphi} \right) \pi\left( \boldsymbol{\theta} \right) \right\}^{\beta} d\boldsymbol{\theta}}$$

$$={{p\left( \boldsymbol{\theta}|\boldsymbol{\varphi},\boldsymbol{X} \right)}^{\beta}}/{\int{p\left( \boldsymbol{\theta}|\boldsymbol{\varphi},\boldsymbol{X} \right)}^{\beta} d\boldsymbol{\theta}}. \left( A5 \right)$$

Thus, from Equation (A5), we obtained the result that the posterior probability of estimates other than modes become zeros for large $\beta\left( \to\infty\right)$. For simplicity, consider $\boldsymbol{\theta}$ has only three values $\left\{ \theta_{1},\theta_{2},\theta_{3} \right\}$, and their posterior probabilities have $p\left( \theta_{1}|\boldsymbol{\varphi},\boldsymbol{X} \right)$ = 0.5, $p\left( \theta_{2}|\boldsymbol{\varphi},\boldsymbol{X} \right)$ = 0.4, and $p\left( \theta_{3}|\boldsymbol{\varphi},\boldsymbol{X} \right)$ = 0.1. The $\beta$-dependent posterior probabilities are easily obtained in this situation:

$$p_{\beta}\left( \theta_{1}|\boldsymbol{\varphi},\boldsymbol{X} \right)=\frac{{p\left( \theta_{1}|\boldsymbol{\varphi},\boldsymbol{X} \right)}^{\beta}}{{p\left( \theta_{1}|\boldsymbol{\varphi},\boldsymbol{X} \right)}^{\beta}+{p\left( \theta_{2}|\boldsymbol{\varphi},\boldsymbol{X} \right)}^{\beta}+{p\left( \theta_{3}|\boldsymbol{\varphi},\boldsymbol{X} \right)}^{\beta}},$$

$$p_{\beta}\left( \theta_{2}|\boldsymbol{\varphi},\boldsymbol{X} \right)=\frac{{p\left( \theta_{2}|\boldsymbol{\varphi},\boldsymbol{X} \right)}^{\beta}}{{p\left( \theta_{1}|\boldsymbol{\varphi},\boldsymbol{X} \right)}^{\beta}+{p\left( \theta_{2}|\boldsymbol{\varphi},\boldsymbol{X} \right)}^{\beta}+{p\left( \theta_{3}|\boldsymbol{\varphi},\boldsymbol{X} \right)}^{\beta}},$$

$$p_{\beta}\left( \theta_{3}|\boldsymbol{\varphi},\boldsymbol{X} \right)=\frac{{p\left( \theta_{3}|\boldsymbol{\varphi},\boldsymbol{X} \right)}^{\beta}}{{p\left( \theta_{1}|\boldsymbol{\varphi},\boldsymbol{X} \right)}^{\beta}+{p\left( \theta_{2}|\boldsymbol{\varphi},\boldsymbol{X} \right)}^{\beta}+{p\left( \theta_{3}|\boldsymbol{\varphi},\boldsymbol{X} \right)}^{\beta}}.$$

If $\beta=10$, $p_{\beta}\left( \theta_{1}|\boldsymbol{\varphi},\boldsymbol{X} \right)=0.903$, $p_{\beta}\left( \theta_{2}|\boldsymbol{\varphi},\boldsymbol{X} \right)=0.097$ and $p_{\beta}\left( \theta_{3}|\boldsymbol{\varphi},\boldsymbol{X} \right)=0.000.$ If $\beta=1000$, they become approximately 1.000, 0.000 and 0.000, respectively.

Our approach is closely related to previous methods of obtaining maximum likelihood estimates (prior feedback, Robert 1993; data cloning, Lele et al. 2007). The difference from their methods is that prior information is considered in $\beta$-dependent posterior distribution. It is noteworthy that data cloning is applied to diagnose underdetermination (referred to as estimability) of maximum likelihood estimates in the same way as our approach (Lele et al. 2010). The concept of $\beta$-dependency (referred to as inverse temperature) is also used in a long-established probabilistic technique for exploring global optimum solutions (simulated annealing, Kirkpatrick et al. 1983).

**S4. Simulation design of our toy example**

Here we describe the design details of our toy simulation: a two-isotope simulation where consumer signature is on the centroid of four source signatures (Fig. 1 in the main text; Fry 2013). The four sources are corners of a square in the mixing space consisting of two isotopes. Our generated model is expressed by Equation (A3). Without loss of generality, we set simulation parameters to the following values:

a. Relative contribution (of the $k$th dietary source):

$$\boldsymbol{f}=\left[ \begin{matrix} \begin{matrix} 0.25 & 0.25 \end{matrix} & \begin{matrix} 0.25 & 0.25 \end{matrix} \end{matrix} \right],$$

b. Source signature mean (of the $k$th dietary source on the $j$th isotope):

$\boldsymbol{\mu}=\left[ \begin{matrix} \begin{matrix} -1 & -1 \\ 1 & -1 \end{matrix} & \begin{matrix} 1 & 1 \\ 1 & -1 \end{matrix} \end{matrix} \right],$

c. Trophic discrimination factor mean (of the $k$th dietary source on the $j$th isotope):

$\boldsymbol{\lambda}=\left[ \begin{matrix} \begin{matrix} 0 & 0 \\ 0 & 0 \end{matrix} & \begin{matrix} 0 & 0 \\ 0 & 0 \end{matrix} \end{matrix} \right],$

d. Source signature variance (of the $k$th dietary source on the $j$th isotope):

$\boldsymbol{\omega}^{\boldsymbol{2}}=\left[ \begin{matrix} \begin{matrix} {0.25}^{2} & {0.25}^{2} \\ {0.25}^{2} & {0.25}^{2} \end{matrix} & \begin{matrix} {0.25}^{2} & {0.25}^{2} \\ {0.25}^{2} & {0.25}^{2} \end{matrix} \end{matrix} \right],$

e. Trophic discrimination factor variance (of the $k$th dietary source on the $j$th isotope):

$\boldsymbol{\tau}^{\boldsymbol{2}}=\left[ \begin{matrix} \begin{matrix} 0 & 0 \\ 0 & 0 \end{matrix} & \begin{matrix} 0 & 0 \\ 0 & 0 \end{matrix} \end{matrix} \right],$

f. Concentration (of the $j$th isotope in the $k$th dietary source):

$\boldsymbol{q}=\left[ \begin{matrix} \begin{matrix} 1 & 1 \\ 1 & 1 \end{matrix} & \begin{matrix} 1 & 1 \\ 1 & 1 \end{matrix} \end{matrix} \right],$

g. Additional unexplained variance (on the $j$th isotope):

$\boldsymbol{\sigma}^{\boldsymbol{2}}=\left[ \begin{matrix} {0.3}^{2} & {0.3}^{2} \end{matrix} \right].$

Then, we used $\boldsymbol{X}=\left[ \begin{matrix} 0 & 0 \end{matrix} \right]$ as an observed isotope signature of consumer individuals. This setting allows us to calculate the rigorous and straightforward solution of our estimation. The variations of simulation parameters do not affect our results and conclusion qualitatively.

**S5. Correlation plots of estimated relative contributions**

This section provides the correlation plots of posterior relative contributions for our toy example (Fig. S1) and published geese data (Fig. S2). Source contributions may be highly correlated when the underdetermined problem exists in the focal mixing space (Semmens et al. 2013).


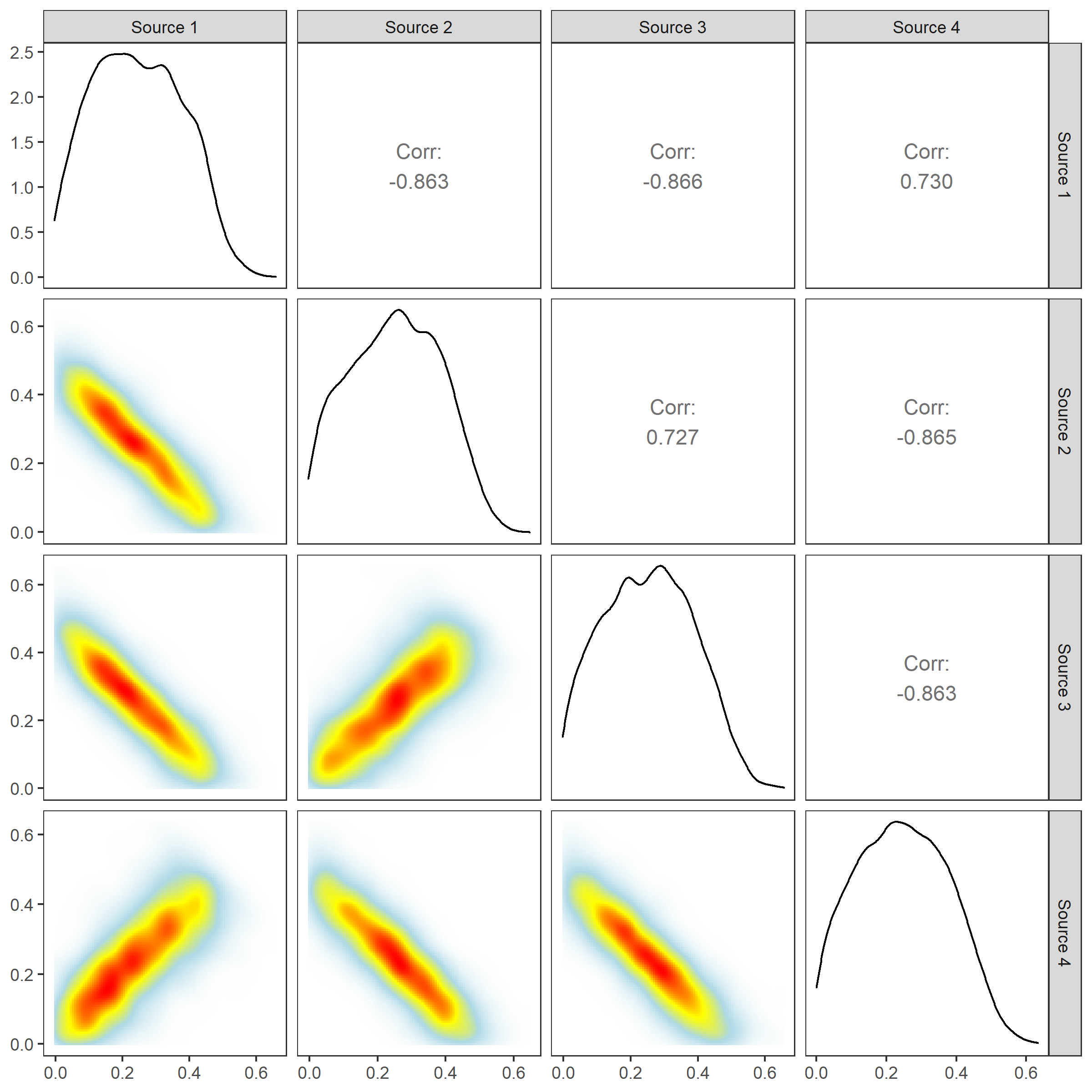


**Figure S1** Correlation plots of posterior relative contributions for toy simulations. The diagonal and lower panels represent marginal and joint posterior distributions, respectively. The joint posterior probability increases from blue to yellow to red.


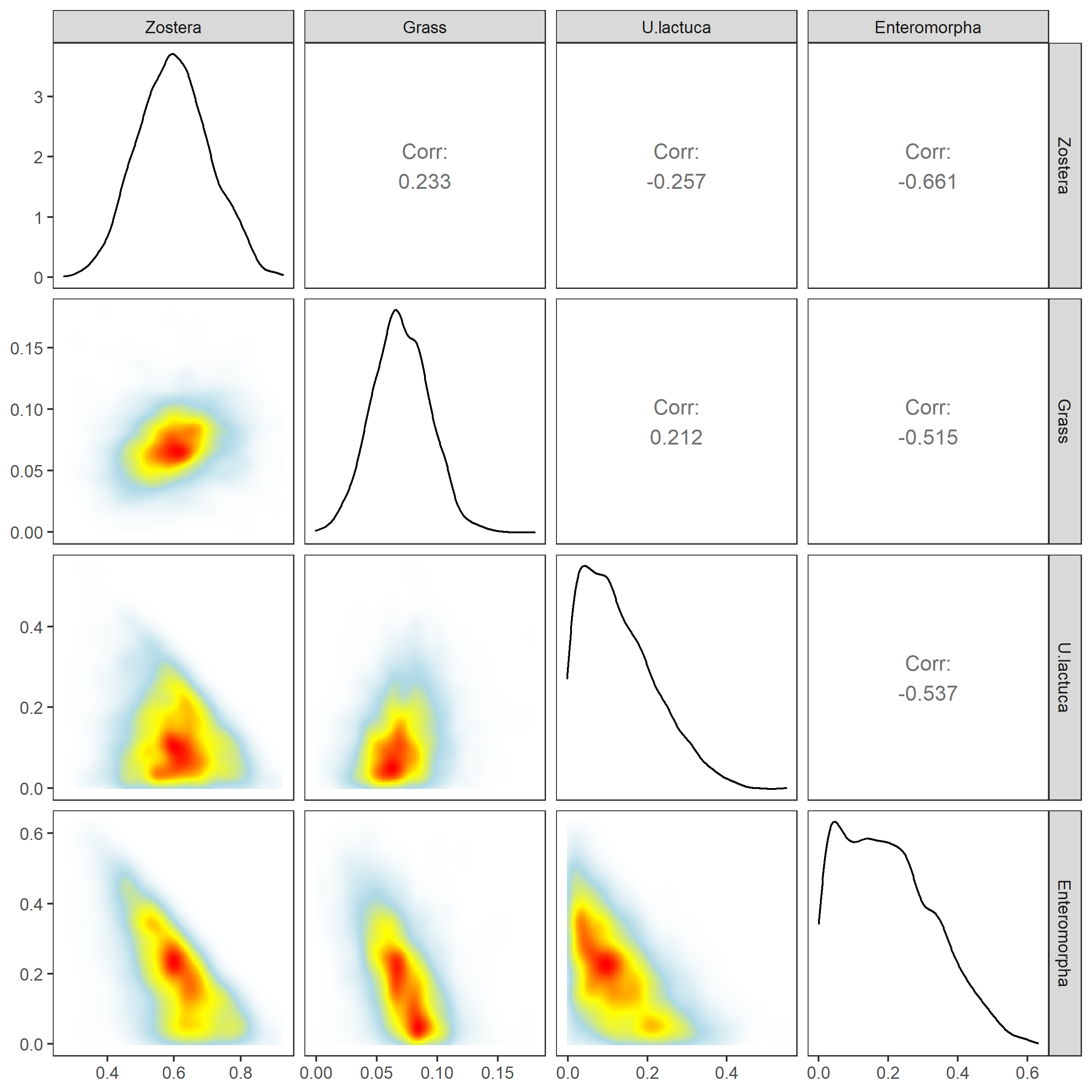


**Figure S2** Correlation plots of posterior relative contributions for published geese data. The diagonal and lower panels represent marginal and joint posterior distributions, respectively. The joint posterior probability increases from blue to yellow to red.

**S6. Additional analysis about different error structure parameterization**

The recent publications (Stock and Semmens 2016; Stock et al. 2018) introduced several parameterizations in the error structure and found that SIAR parameterization may poorly perform than other parameterizations by comparing DIC. To investigate the influence of error structure parameterizations on underdetermination, we re-analyze published geese data by modeling the following error structure (i.e., Equation 4 in Stock and Semmens 2016):

$$p\left( \boldsymbol{X}|\boldsymbol{\theta,\varphi} \right)=p\left( \boldsymbol{X}|\boldsymbol{f},\boldsymbol{q},\boldsymbol{\mu},\boldsymbol{\omega},\boldsymbol{\lambda},\boldsymbol{\tau},\boldsymbol{\varepsilon} \right)$$

$$=\prod_{i=1}^{N} \prod_{j=1}^{J} N\left( X_{ij} | \frac{\sum_{k=1}^{K} f_{k}q_{jk}\left( \mu_{jk}+\lambda_{jk} \right)}{\sum_{k=1}^{K} f_{k}q_{jk}}, \frac{\sum_{k=1}^{K} {f_{k}}^{2}{q_{jk}}^{2}\left( {\omega_{jk}}^{2}+{\tau_{jk}}^{2} \right)}{\left( \sum_{k=1}^{K} f_{k}q_{jk} \right)^{2}}\times\varepsilon_{j} \right)$$

$$\pi\left( \boldsymbol{\theta} \right)=\pi\left( \boldsymbol{f} \right) \pi\left( \boldsymbol{\sigma} \right)=Dir\left( \boldsymbol{f} | \boldsymbol{a} \right) \left\{ \prod_{j=1}^{J} U\left( \varepsilon_{j} | 0, 20 \right) \right\}.$$

where $\boldsymbol{\varepsilon}$ is used as an error parameterization instead of $\boldsymbol{\sigma}^{\boldsymbol{2}}$ of SIAR. We found that Stock’s parameterization leads to wider ΔBDP than that of SIAR but improves the consistency between ordinary and beta-dependent marginal posteriors (Table S1, Fig. S3). This result suggests that underdetermination may be unexpectedly influenced by slight difference of model structures.


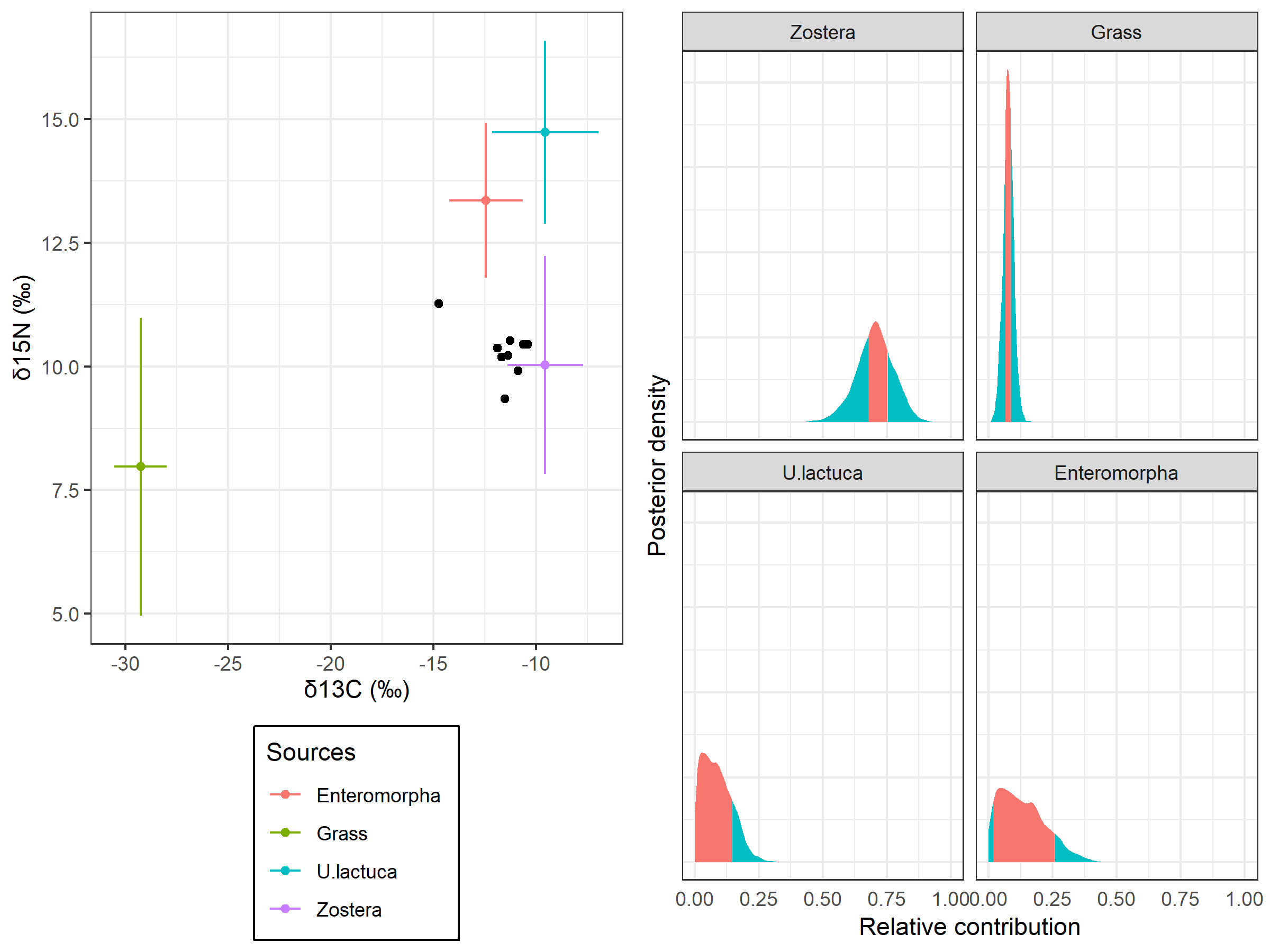


**Figure S3** Isotopic mixing space and model results of published Brent geese data. Figure descriptions are the same as Fig. 3 in the main text. The difference between this figure and Fig. 3 is the error structure parameterization of SIMMs.

**Table S1** Estimated relative contributions (%) of dietary sources for published geese data. The medians, 95% credible intervals (CI) and mean determination coefficient ($\bar{r}^{2}$), BDPs and their widths (ΔBDP) are calculated. We set *β* = 1,000 for *β*-dependent SIAR.

|  | medians | 95% CI | $\bar{r}^{2}$ | BDP | ΔBDP |
| --- | --- | --- | --- | --- | --- |
| *Zostera* | 70.4 | [54.9, 83.8] | 0.167 | [67.7, 75.2] | 7.5 |
| Terrestrial grasses | 7.6 | [ 3.6, 11.8] | 0.068 | [ 6.4, 8.7] | 2.3 |
| *Ulva lactuca* | 8.0 | [ 0.5, 21.6] | 0.155 | [ 0.0, 14.6] | 14.6 |
| *Enteromorpha* | 12.0 | [ 0.6, 33.7] | 0.300 | [ 1.8, 26.0] | 24.2 |

**References**

Phillips, D. L. 2001. Mixing models in analyses of diet using multiple stable isotopes: a critique. Oecologia, 127, 166-170.

Phillips, D. L. and Gregg, J. W. 2003. Source partitioning using stable isotopes: coping with too many sources. Oecologia, 136, 261-269.

Fry, B. 2013. Alternative approaches for solving underdetermined isotope mixing problems. Marine Ecology Progress Series, 472, 1-13. doi: 10.3354/meps10168

Semmens, B. X., Ward, E. J., Parnell, A. C., Phillips, D. L., Bearhop, S., Inger, R., … Moore, J. W. 2013. Statistical basis and outputs of stable isotope mixing models: comment on Fry (2013). Marine Ecology Progress Series, 490, 285-289.

Brett M. T. 2014. Resource polygon geometry predicts Bayesian stable isotope mixing model bias. Marine Ecology Progress Series, 514, 1-12.

Stock, B. C., Jackson A. L., Ward E. J., Parnell, A. C., Phillips D. L. and B. X. Semmens 2018. Analyzing mixing systems using a new generation of Bayesian tracer mixing models. PeerJ, 6, e5096.

Parnell, A. C., Inger, R., Bearhop, S. and Jackson, A. L. 2010. Source partitioning using stable isotopes: coping with too much variation. Plos One, 5, e9672.

Robert C. P. 1993. Prior feedback: a Bayesian approach to maximum likelihood distribution. Computational Statistics 8. 279-294.

Lele, S. R., Dennis, B. and Lutscher, F. 2007. Data cloning: easy maximum likelihood estimation for complex ecological models using Bayesian Markov chain Monte Carlo methods. Ecology Letters, 10, 551-563.

Lele, S. R., Nadeem, K. and Schmuland, B. 2010. Estimability and likelihood inference for generalized linear mixed models using data cloning. Journal of the American Statistical Association, 105, 1617-1625.

Kirkpatrick, S., Gelatt, C. D. and Vecchi, M. P. 1983. Optimization by simulated annealing. Science, 220, 671-680.

Stock, B. C. and Semmens B. X. 2016. Unifying error structures in commonly used biotracer mixing models. Ecology, 97, 2562-2569.
